# A de novo transcriptome of the noble scallop, *Chlamys nobilis*, focusing on mining transcripts for carotenoid-based coloration

**DOI:** 10.1186/s12864-015-1241-x

**Published:** 2015-02-05

**Authors:** Helu Liu, Huaiping Zheng, Hongkuan Zhang, Longhui Deng, Wenhua Liu, Shuqi Wang, Fang Meng, Yajun Wang, Zhicheng Guo, Shengkang Li, Guofan Zhang

**Affiliations:** Key Laboratory of Marine Biotechnology of Guangdong Province, Shantou University, Shantou, 515063 China; Department of Education of Guangdong Province, Mariculture Research Center for Subtropical Shellfish & Algae, Shantou, 515063 China; Sanya Institute of Deep-sea Science and Engineering, Chinese Academy of Science, Sanya, 572000 China; Institute of Oceanology, Chinese Academy of Sciences, Qingdao, 266071 China

**Keywords:** *Chlamys nobilis*, Transcriptome sequencing, Carotenoid coloration, Candidate genes

## Abstract

**Background:**

The noble scallop *Chlamys nobilis* Reeve displays polymorphism in shell and muscle colors. Previous research showed that the orange scallops with orange shell and muscle had a significantly higher carotenoid content than the brown ones with brown shell and white muscle. There is currently a need to identify candidate genes associated with carotenoid-based coloration.

**Results:**

In the present study, 454 GS-FLX sequencing of noble scallop transcriptome yielded 1,181,060 clean sequence reads, which were assembled into 49,717 isotigs, leaving 110,158 reads as the singletons. Of the 159,875 unique sequences, 11.84% isotigs and 9.35% singletons were annotated. Moreover, 3,844 SSRs and over 120,000 high confidence variants (SNPs and INDELs) were identified. Especially, one class B scavenge receptor termed SRB-like-3 was discovered to express only in orange scallops and absent in brown ones, suggesting a significant association with high carotenoid content. Down-regulation of SRB-like-3 mRNA by RNA interference remarkably decreased blood carotenoid, providing compelling evidence that SRB-like-3 is an ideal candidate gene controlling carotenoid deposition and determining orange coloration.

**Conclusion:**

Transcriptome analysis of noble scallop reveals a novel scavenger receptor significantly associated with orange scallop rich in carotenoid content. Our findings pave the way for further functional elucidation of this gene and molecular basis of carotenoid deposition in orange scallop.

**Electronic supplementary material:**

The online version of this article (doi:10.1186/s12864-015-1241-x) contains supplementary material, which is available to authorized users.

## Background

Carotenoids are bright yellow and red pigments that are responsible for some coloration found in animals [1]. Carotenoids also play important physiological roles such as acting as antioxidants in the immune system [2,3]. Unlike other pigments types such as melanins, carotenoids cannot be synthesized by animals and must be acquired through diet [4]. There are a number of factors (such as food source, seasonal change) that potentially limit the ability of animals to deposit carotenoids in their body tissues [5,6]. Although carotenoid traits have often been shown to be condition-dependent, carotenoid coloration and accumulation is also dependent on underlying genetic mechanisms. Animals preferentially deposit certain carotenoids over others, and are able to enzymatically convert and cleave dietary carotenoids into other derived forms [7], implying strongly the involvement of genes encoding appropriate carotenoid-binding and transport proteins or enzymes participating carotenoid metabolism.

The SRB (scavenger receptor class B) is first identified as playing a role in the uptake of lutein [8], carotene [9], zeaxanthin and xanthophylls [10], and lycopene [11]. A SRB homologue, *ninaD*, is essential for cellular uptake of carotenoids in *Drosophila* and a mutation in this gene results in carotenoid-free and thus a vitamin A deficient phenotype [12]. Two recently cloned genes, Cameo2 and SCRB15 of CD36 (Cluster Determinant 36), which are homologous to SRB, have been shown to be involved in the selective transport of lutein and β-carotene, respectively, into the silk gland of *Bombyx mori* [13,14]. StAR (steroidogenic acute regulatory)/MLN64 (metastatic lymph node 64) are members of the StAR domain family that are involved in the intracellular transport of cholesterol for the initiation of steroidogenesis [15]. StAR isolated in the macula of primate retina could selectively bind lutein with high affinity [16]. The *B. mori* carotenoid-binding protein (CBP) is an orthologue of vertebrate MLN64, and is involved in the transport of lutein [17]. The BCMO (β,β-carotene-15,15’-monooxygenase)/BCDO(β,β-carotene-9,9’-oxygenase) is involved in the enzymatic cleavage of carotenoids [18]. Loss-of-function mutation in BCMO results in hypercarotenemia [19]. Carotenoids are cleaved to form colorless apo-carotenoid derivatives in chickens with white skin, while yellow-skinned chickens presumably have one or more cis-acting regulatory mutations in BCDO, resulting in a yellow coloration in the skin because of deposition of uncleaved carotenoids [20]. Other genes involved in the transport and binding of carotenoids are Niemann Pick C1-like 1 (*NPC1L1*) [21], ATP-binding cassette sub-family G member 5 (ABCG5) [22], Glutathione S-transferase Pi1 (GST) [23] and crustacyanin [24]. Lastly, intestinal transcription factor (ISX) [25] and retinoic acid receptor (RAR)/retinoid X receptor (RXR) [26] are important transcription factors that regulate the expression of genes (such as BCMO and SRB) involved in carotenoid deposition.

The noble scallop *Chlamys nobilis* Reeve, an important aquaculture bivalve in China, displays conspicuous polymorphism in shell color (such as orange, orange-purple, brown, etc.) and difference in muscle color (such as orange and white). The orange scallops have carotenoid-based orange mantle and adductor muscle due to high presence of carotenoids. Our previous work showed that the orange scallops with orange shell and muscle had a significantly higher carotenoid content than the brown ones with brown shell and white muscle [27]. By establishing different scallop lines, both shell color and muscle color have been confirmed to be control by at least two loci, with one locus showing dominance epistasis to the other [28,29]. Therefore, the carotenoid-based orange coloration in muscle is likely due to differential expression of one or a few genes at the site of carotenoid deposition.

In recent years, transcriptome analysis has been widely recognized as a very useful tool to identify candidate genes underlying molecular mechanisms. In the present study, we first sequenced and assembled the transcriptome of noble scallop *C. nobilis* using a GS-FLX 454 platform. Second, we quantified the expression of genes that are homologous to known carotenoid candidate genes in the adductor muscle, which actively deposits carotenoids in scallop, and investigated whether differential expression was associated with carotenoid content variation in orange scallops versus brown scallops. Our goals were to 1) generate a transcriptome database useful for functional genetic studies of *C. nobilis*; and 2) identify candidate transcripts involved in carotenoid-based coloration or carotenoid deposition.

## Methods

### Ethics statement

The scallops used in this study were taken from Nan’ao Marine Biology Station of Shantou University, located at Nan’ao island of Shantou, Guangdong, China. No specific permits were required for the described field studies, as the sampling locations were not privately owned or protected in any way. These field studies also did not include endangered or protected species. The animals were processed according to “the Regulations for the Administration of Affairs Concerning Experimental Animals” established by the Guangdong Provincial Department of Science and Technology on the Use and Care of Animals.

### Sample collection and preparation

**Figure 1:**
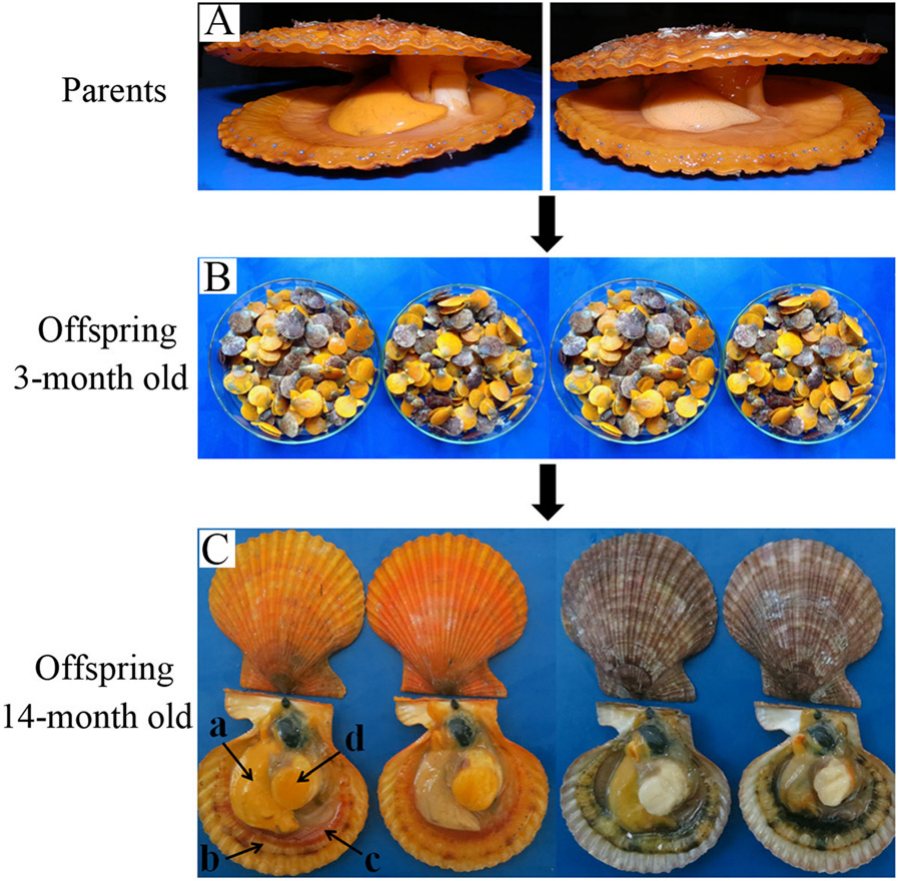
**Line of the** ***C. nobilis*** **was used in transcriptome sequencing. (A)** Both parents are orange scallop. **(B)** Offspring with orange and brown coloration segregation obtained by crossing two orange scallops. **(C)** Four kinds of tissues: gonad (a), mantle (b), gill (c), and adductor muscle (d). Male scallop has a white or lighter orange color gonad, while female scallop has a heavier orange color gonad.

In noble scallop, orange color was dominant to brown color. Color segregation occurred when crossing two orange scallops [28,29]. Both orange and brown scallops used in the present study were from a line of F_2_ generation produced by continuous crossing orange parental scallops (Figure 1A). A total of 20 orange scallops (rich in carotenoids) and 20 brown scallops (lack of carotenoids) at 14-month old were randomly chosen. Average shell size in length, height and width for the orange was 67.28 ± 4.22 mm, 72.92 ± 3.73 mm and 24.06 ± 1.66 mm, and for the brown was 66.80 ± 3.09 mm, 71.69 ± 3.73 mm and 23.88 ± 1.15 mm, respectively. Tissues including the gonad, mantle, gill and adductor muscle (Figure 1C) were sampled and homogenized with the QiaShredder (Qiagen, Germany) for total RNA extraction using the Qiagen RNeasy (Qiagen, Germany) kit. mRNA was then purified by using the Qiagen Oligotex mRNA purification kit. Equal amounts of mRNA from four tissues were pooled for either an “orange” or a “brown” scallop sample. From the two pooled samples, about 600 ng mRNA was used for cDNA generation with the SMART cDNA synthesis kit (Clontech Laboratories, USA). Quality control in each extraction step was investigated using gel electrophoresis and nanodrop spectrophotometry (Peqlab, Germany). Both orange and brown scallop cDNAs were further checked with a Bioanalyzer 2100 (Agilent Technologies, USA). As a result, two cDNA libraries (one for orange scallop and the other for brown scallop) with an average length of 400 bp were generated according to the manufacturers’ protocol and sequenced on a 454 Genome Sequencer system (Roche Life Sciences, USA) with FLX and Titanium chemistry.

### Sequence assembly and functional annotation

All sequence reads taken directly from the 454 GS-FLX sequencer were run through the sff file program (Newbler v2.6, Roche) to remove sequencing adapters A and B. Barcodes were removed by Seqclean (Lastest86_64) program and poor sequence data were further cleaned by Lucy v1.20 program (–m 50 –e 0.03 0.03 –w 30 0.03 10 0.1 –b 4 0.03). Sequences with homopolymers of a single nucleotide occupying 60% of the read and those less than 50 nucleotides in length were discarded. Trimmed sequences from orange or brown scallop were mixed and then assembled *de novo* using the default parameters of Newbler v2.6 (Roche). All *C. nobilis* EST (expressed sequence tags) sequences were submitted to NCBI Sequence Read Archive under Accession No. SRX253988. ESTs that did not form isotigs (singletons) and isotigs resulting from the assembly of multiple sequences were referred to as unique sequences. These unique sequences were translated into six reading frames and used as a query to search the public databases including Non-redundant protein database (Nr) and Swiss-Prot database (Swiss-Prot). All unique sequences were sequentially compared using BlastX (cut-off E-value of 1e^-5^) with the sequences in two public protein databases (Nr and Swiss-Prot). Once a sequence had a blast hit in one of the databases, a description was built from the description of that hit. Additionally, Gene Ontology (GO) terms were deduced from the blast results using Blast2GO, and sorted into the immediate subcategories for ‘molecular function’, ‘cellular component’ and ‘biological process’.

### Identification of EST-SSR motifs and EST-SNPs

All EST sequences were searched for SSR motifs using the MISA (MIcroSAtellite identification) program (http://pgrc.ipk-gatersleben.de/misa/). Default settings were employed to detect perfect di-, tri-, tetra-, penta-, and hexa-nucleotide motifs (including compound motifs). To be assigned, di-nucleotide SSRs (Simple Sequence Repeats) required a minimum of 6 repeats, and all other SSR types needed a minimum of 5 repeats. Two neighboring SSRs with the maximum interruption no more than 100 nucleotides were considered as a compound SSR.

Multiple nucleotide sequence alignments of isotigs identified among the EST libraries were undertaken to identify putative SNPs. Since few reference sequences were available, SNPs were identified as superimposed nucleotide peaks where 2 or more reads contained polymorphisms at the variant allele. SNPs were identified using default parameters in gsMapper v2.3 (Roche) to align isotigs from two color datasets. In addition, only an overall transition vs transversion (Ts/Tv) ratio was calculated across the dataset. Perl script modules linked to the primer modeling software Primer3 were used to design PCR primers flanking for each unique SNP region identified.

### Data mining of transcripts with putative function involved in carotenoid Deposition

From public databases, we compiled a dataset of the 15 known gene involved in carotenoid deposition were collected (Table 1). The amino-acid sequences of the known carotenoid deposition genes, covering carotenoid absorption, transport and cleavage, were used to search (tBlastn) for homologues in 454-derived sequences. Those sequences with scores more than or equal to 100 and E values less than or equal to 1e-10 were clustered to develop unigenes, and all of the unigenes were considered as candidate transcripts. The resulting unigenes were in turn used to search the GenBank databases by BlastX to confirm their putative carotenoid-related functions.

**Table 1:** **Carotenoid-related candidate gene**

**Name**	**Evidence for potential role in carotenoid deposition**	**Protein ID**	**Reference**
ninaD	Responsible for carotenoid uptake in *Drosophila* and mutation leads to carotenoid deficient	AAO11676	[12]
SRB type I	Involved in the uptake of carotenoids; homologous to ninaD in *Drosophila*	NP_005496	[10]
Cameo2	Involved in selective absorption of lutein	BAI66272	[13]
CD36	Homologous to carotenoid-uptake gene Cameo2 and SCRB15 in *Bombyx mori*	NP_000063	[13,14]
Carotenoid binding protein (CBP)	Involved in lutein binding and transportation in *B. mori*	BAC01051	[17]
MLN64(STAR3)	Orthologue of *B. mori* CBP	NP_001159410	[16]
Crustacyanin	CBP in the carapace of crustaceans and binding astaxanthin	1GKA_B	[24]
BCDO	Lower expression levels lead to the retention of carotenoids and a yellow skin phenotype	ACA05952	[18,19]
BCMO	Lower activity leads to hypercarotenemia in human being	NP_059125	[20]
NPC1L1	Involved in intocopherol intestinal absorption using Caco-2 cells and in situ perfusions in rats. Lower expression levels inhibit the uptake of several carotenoids in Caco-2 cells.	NP_037521	[21]
GST	Binding carotenoid in the mammalian retina	AAH10915	[23]
ABCG5	A genetic variant in ABCG5 associate with plasma lutein concentration	NP_071881	[22]
RXR/RAR	Form heterodimers of RXR-RAR and regulate retinoid-responsive elements	RXR:NP_002948	[26]
		RAR:NP_000955	
ISX	Gatekeeper that controls intestinal β, β-carotene absorption	NP_082113	[25]

### mRNA expression of selected candidate transcripts in orange and brown scallop

Expression of selected transcripts was investigated in adductor muscle from 6 orange scallop or 6 brown scallops at 14-month old, and two technical replicates were performed for each scallop. All scallops used in this experiment were from a F_2_ generation as described above, and cultured in the same cage. Total RNA was extracted and quality and quantity determined using a nanodrop spectrophotometer. 1 μg mRNA was used to synthesize cDNA by PrimeScript RT reagent kit with gDNA Eraser (TaKaRa). Quantitative real-time RT-PCR was conducted in a LightCycler®480 System using the SYBR Premix Ex Taq II qRT-PCR Kit (TaKaRa). Each assay was performed with *β*-actin mRNA as the internal control. The real-time PCR program was 95°C for 30s, followed by 40 cycles of 95°C for 5 s, and 60°C for 30s according to the instructions of the manufacturer. Dissociation analysis of amplification products was performed at the end of each PCR reaction to confirm that only one PCR product was amplified and detected. The comparative CT method (2^-ΔΔCT^ method) was used to analyze the expression level of each candidate genes. All data were given in terms of relative mRNA expressed as means ± SE. The data were subjected to analysis of one-way ANOVA, and p-values smaller than 0.05 were considered statistically significant.

### Detecting presence of SRB (scavenger receptor class B)-3-like and measurement of total carotenoid content in scallops

Four scallop lines derived from orange parents, which have color segregation of orange and brown, were chosen to performed this experiment. In total, 80 scallops (40 orange and 40 brown), derived from 4 lines produced by crossing two orange scallops in the Spring of 2012, were used to detect the presence of SRB-3-like in the blood and determine total carotenoid content in the adductor muscle. Presence of SRB-3-like in the blood was detected using primers S3F1: CGATTTTGGAACGGTAACAGTAACTTGGA and S3R1: ATGGATTGACTGATGTGAGATGT. PCR amplification product was confirmed by sequencing. Total carotenoid content in the adductor muscle was determined using the method of Zheng et al. [27].

### dsRNA synthesis

SRB-like-3 gene was amplified through PCR with noble scallop cDNA as template and 1Fi and 1Ri as primers (Table 2). The PCR products were separated, purified, ligated with vector pMD-18 T (Takara), and transformed into DH5α *E. coli* cell. The plasmid was extracted using MiniBEST Plasmid Purification Kit Ver.4.0 (Takara) according to the manufacturer’s protocol.

**Table 2:** **Primer sequences used for dsRNA synthesis**

**Primer**	**Sequence**
1Fi	CGATTTTGGAACGGTAACAGTAACTTGGA
1Ri	ATGGATTGACTGATGTGAGATGT
2Fi	GATCACtaatacgactcactatagggAACGGTAACAGTAACTTGGA
2Ri	GATCACtaatacgactcactatagggTGAGATGTTTGATGATTTCCGTA
EGFPF	GATCACtaatacgactcactatagggCAGTGCTTCAGCCGCTACCC
EGFPF	GATCACtaatacgactcactatagggAGTTCACCTTGATGCCGTTCTT

Note: The lower case is T7 promoter sequence.

For dsRNA synthesis, SRB-like-3 was amplified by PCR with the primers 2Fi and 2Ri (containing T7 promoter) using the recombinant plasmid pMD-18 T-SRB as the template and (Table 2). Similarly, for dsRNA synthesis of EGFP gene [30], plasmid pEGFP-N1 was used as the template for PCR using EGFPF and EGFPR as the primers. Quantity and quality of the DNA fragments were assessed by nanodrop spectrophotometry and electrophoresis in 1.0% agarose gel. dsRNA was synthesized in vitro using MEGAscript RNAi Kit (Life Technology) following the manufacturer’s protocol. After being incubated at 75°C for 5 min, dsRNA was cooled to room temperature, digested with DNase and RNase, and purified.

### RNAi (RNA interference) assay

Forty orange scallops were used, and each of them was injected with 40 μg dsRNA of SRB-like-3 or EGFP gene (as a control) into the adductor muscle. Scallops were labeled and placed in a cage. The blank group was injected with Rnase-free water. Five individuals were sampled at 3, 6, 12, and 24 h for each group. Adductor muscle muscle, blood and intestine were subjected to total RNA extraction. Real-Time PCR was performed as described above with 2 technological replicates for each sample.

### Effect of dsRNA on carotenoid deposition in the blood and adductor muscle

Orange scallops were randomly chosen, and 20 of them were injected with dsRNA of SRB-like-3 or EGFP gene, 5 of them were injected with RNA-free water as the blank group. 24 h later, they were injected again. 12 h after the second injection, 5 scallops from Rnase-free water group, 10 scallops from dsSRB-like-3 group, and 10 scallops from dsEGFP group were sampled. 1 ml blood from each scallop was freeze-dried and added with 0.5 ml acetone to extract caroteoid for about 2-4 h at darkness. Caroteoids from adductor muscle were extracted according to method by Zheng et al. [27]. The samples were always under N_2_ until measurement of absorption at 480 nm to determine their carotenoid content.

## Results and discussion

### Roche 454 GS-FLX sequencing and isotigs assembly

**Figure 2:**
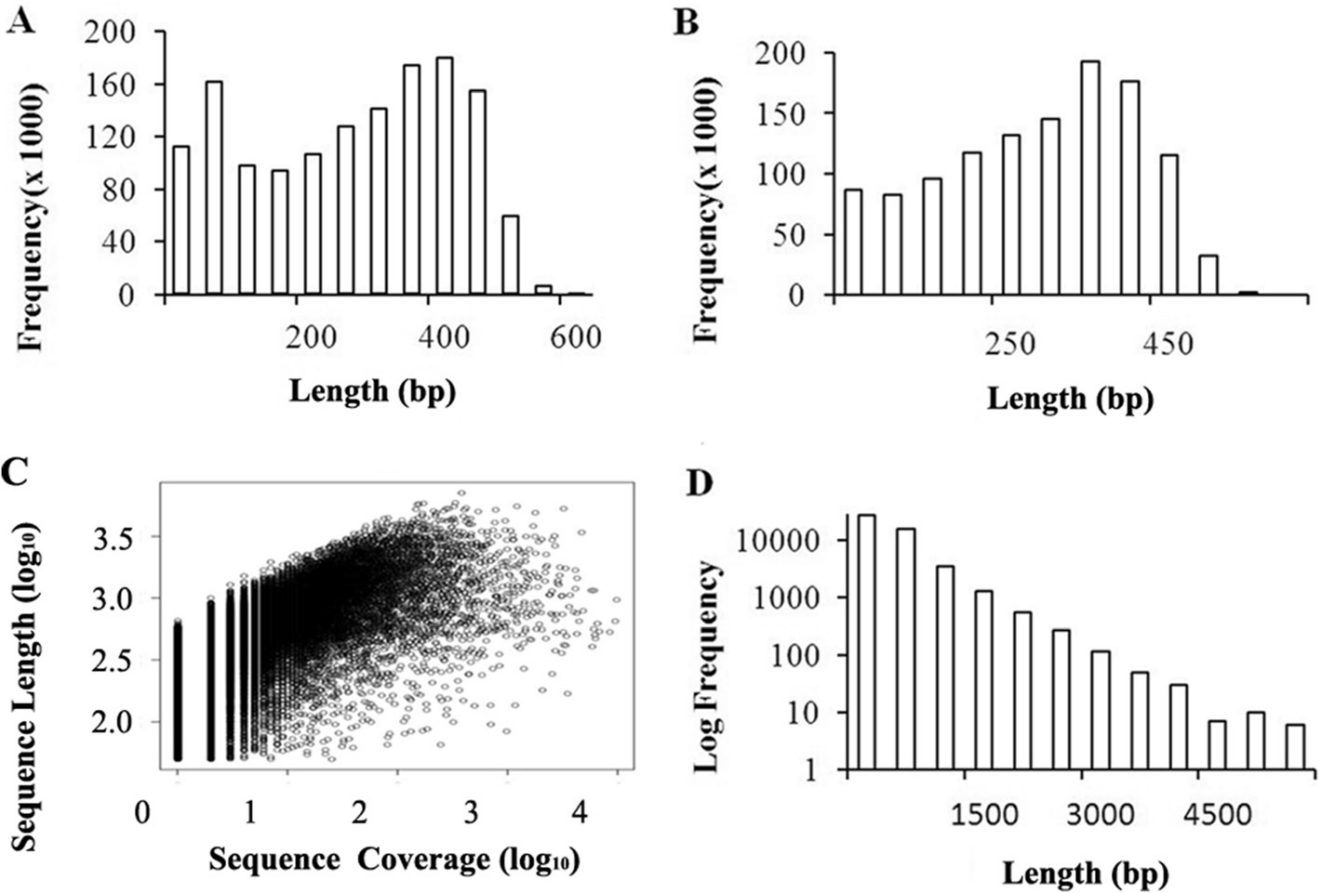
**Overview of the** ***C. nobilis*** **transcriptome sequencing and assembly. (A)** Size distribution of 454 raw reads. **(B)** Size distribution of 454 reads after removal of adaptor and short sequences. **(C)** Log-log plot showing the dependence of isotigs lengths on the number of reads assembled into each isotigs. **(D)** Size distribution of isotigs.

A total of 1,416,522 raw reads were obtained from the 454 GS-FLX sequencer, including 1,181,060 clean reads (averaging 308 bp in length) after adaptor trimming, size-selection and quality control (Table 3). The size distribution of raw reads and clean reads are shown in Figure 2A and B.

**Table 3:** ***Summary statistics for EST and de novo assembly***

	***Total***	***Brown***	***Orange***
**NO. of raw reads**	1,416,522	610,701	805,821
**Average length (bp)**	282	257	301
**% reads removed**	16.62	25.51	11.05
**NO. of reads after cleaning**	1,181,060	454,933	716,797
**Average length of cleaned reads (bp)**	308	297	310
**NO. of reads assembled as isotigs**	1,070,902	NA	NA
**NO. of isotigs**	49,717	NA	NA
**Average length of isotigs**	580	NA	NA
**Range of isotig lengths**	50 - 7,102 bp	NA	NA
**Isotigs above 200 bp**	43,763	NA	NA
**NO. of singletons**	110,158	NA	NA
**Average length of singletons**	296	NA	NA
**Range of singletons lengths**	50 - 654 bp	NA	NA
**Singletons above 200 bp**	80,501	NA	NA
**NO. of unique sequences** ^**a**^	159,875	NA	NA
**NO. of unique sequences (left after CD-hit)**	111,670	NA	NA
**Unique sequences above 200 bases**	87,120	NA	NA

^a^The total number of isotigs and singletons.

Sequences that passed basic quality standards were clustered and assembled *de novo* (Newbler v2.60; Roche). Overall, approximately 91% (1,070,902) reads were assembled into 49,717 isotigs, and the others (110,158) remained as singletons (Table 1). Sequencing coverage of isotigs is shown in Figure 2C with an average 7-fold coverage. The size distribution of isotigs is shown in Figure 2D, which ranges from 50 to 7,102 bp with an average of 580 bp. The percentage of reads assembled *de novo* is similar to that found in other studies [31-33]. The large numbers of unique sequences (singletons and isotigs) in this study are likely due to the extensive diversity in the initial RNA samples as mentioned above. Different organs and sexes, and sequence variants in individuals are known to produce extensive alternatively spliced transcripts, resulting in misalignments and incorrect assembly between reads arising from the same genomic region [34].

### Annotation of the transcriptome

All isotigs and singletons were subjected to CD-hit program (version 4.5.6) to remove redundant sequences, leaving 111,670 unique sequences (46,284 isotigs, 65,386 singletons). The annotation for unique sequences from *C. nobilis*was based on sequence similarity searches against public databases. These databases included NCBI Nr and SwissProt. About 21.19% of all sequences (11.84% isotigs and 9.35% singletons) identified a homologue mostly in both or at least one of the databases at e-value 1e^-5^ (Additional file 1: Table S1). Because the significance of sequence similarity depends in part on the length of the query sequence, short unique sequences frequently cannot be matched to known genes [31,35]. The proportion of sequences with matches in public databases was greater for the longer assembled sequences. Namely, 24.12% matches were recorded for sequences ≥ 300 bp, 45.29% for those longer than 1 kb, but only 5.79% for those short sequences (<300 bp) (Table 4). The percentage of sequences with annotation information in this study was considerably low (approximately 17.43%). The poor annotation efficiency may be due to the insufficient sequences in public databases for phylogenetically close species to date [31,36].

**Table 4:** **Summary of annotation of the** ***C. nobilis*** **transcriptome**

	***Isotigs (singletons)***	***≥300 bp***	***≥1000 bp***
**Total number of sequences**	46,284 (65,386)	70,930	5,849
**Sequences with Blast matches against Nr database**	13,223 (10,438)	20,434	2,910
**Sequences with Blast matches against SwissProt database**	9,409 (6,648)	14,234	2,353
**Sequences assigned GO terms**	5,360 (4,691)	8,463	1,202
**ESTs assigned with EC numbers**	2,054 (1,221)	2,992	538

### Gene ontology assignments

**Figure 3:**
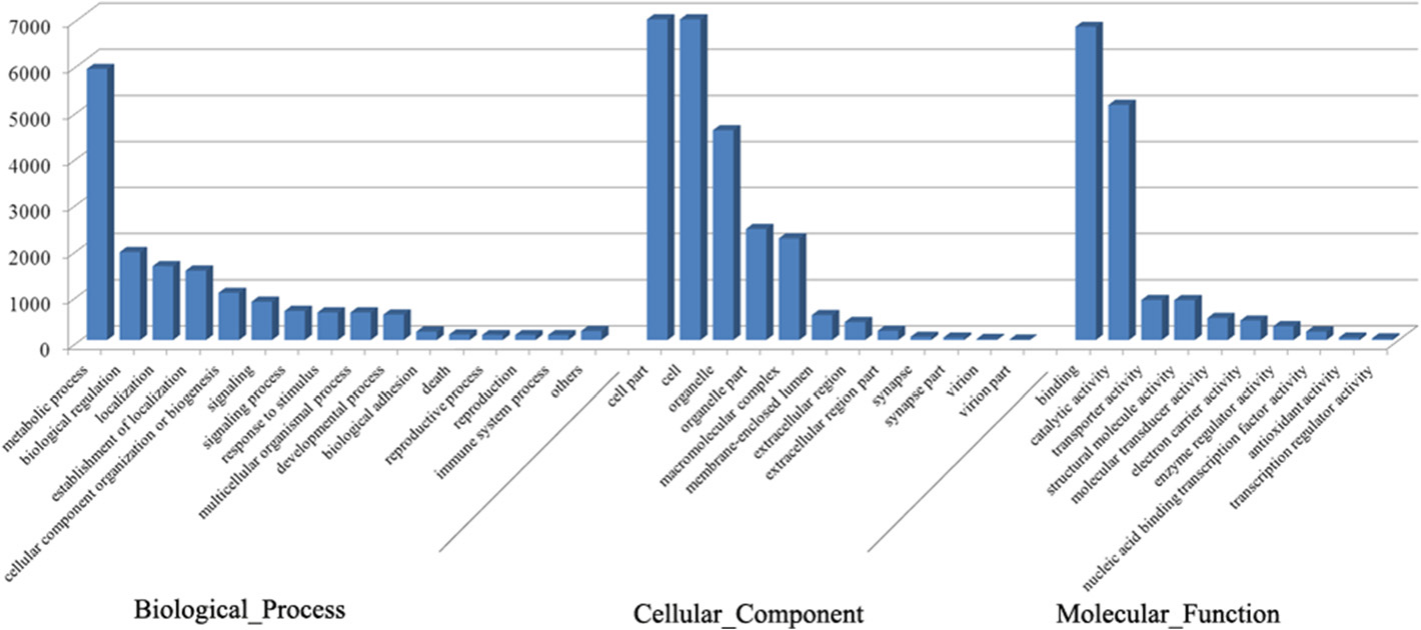
**Functional annotation of assembled sequences based on gene ontology (GO) categorization.** GO analysis was performed at the level 2 for three main categories (cellular component, molecular function and biological process).

Gene Ontology (GO) [37] could provide a dynamic, controlled vocabulary and hierarchical relationships for the representation of information on molecular function, cellular component and biological process, allowing a coherent annotation of gene products. Of annotated unique sequences in Nr or SwissProt database, 10,051 unique sequences were assigned to one or more GO terms (Additional file 2: Table S2). A total of 4,031 GO terms were obtained, with 21,182 unique sequences for biological processes, 24,348 unique sequences for cellular components and 15,122 unique sequences for molecular function (Figure 3). Of those sequences for biological processes, the major ones were cellular process (27.88%) and metabolic process (26.51%). For cellular component, the most represented categories were cell (28.55%) and cell part (28.55%). Regarding molecular functions, binding (44.95%) was the most represented sequences according to GO terms, followed by catalytic activity (33.79%). Similar results were also found in other species such as the Yesso scallop (*Patinopecten yessoensis*) [31] and the freshwater prawn (*Macrobrachium rosenbergii*) [33]. These GO annotations can provide a comprehensive information on transcript functions of *C. nobilis*.

### Putative molecular markers

Selection based on molecular markers is well known to be vital in shellfish aquaculture industry [38]. 454 sequencing may provide an excellent source for mining and development of these markers for *C. nobilis*, because few genetic markers are currently available. A total of 3,259 unique sequences were found to possess 3,479 SSRs, of which 276 (7.93%) unique sequences annotated in Nr and/or Swissport database were considered as priority candidates for maker development (Table 5, Additional file 3: Table S3). The most frequent repeat motifs were di-nucleotide repeats (68.12%), followed by tri-nucleotide repeats (25.29%) and tetra-nucleotide repeats (5.81%). Penta-nucleotides repeats and Hexa-nucleotides repeats only accounted for 0.78%. Among the di-nucleotide repeat classes, TA was the most frequent dimer motif (47.93%). With regard to tri-nucleotide repeats, TGG (6.48%) was the most common motif, followed by GAT (6.02%) and TGA (5.00%).

**Table 5:** **Summary of simple sequence repeat (SSR) nucleotide classes among different nucleotide types found in** ***C. nobilis*** **sequences**

***SSR type***	***No. of SSR- containing ESTs***	***NO. of SSRs***	***% of total SSRs***
**Di-nucleotides**	2,197	2,370	68.12
**Tri-nucleotides**	868	880	25.29
**Tetra-nucleotides**	202	202	5.81
**Penta-nucleotides**	18	18	0.52
**Hexa-nucleotides**	9	9	0.26
**Total**	3,294	3,479	100

Note: Both isotigs and singletons sequences are used to predict the SSR loci.

**Figure 4:**
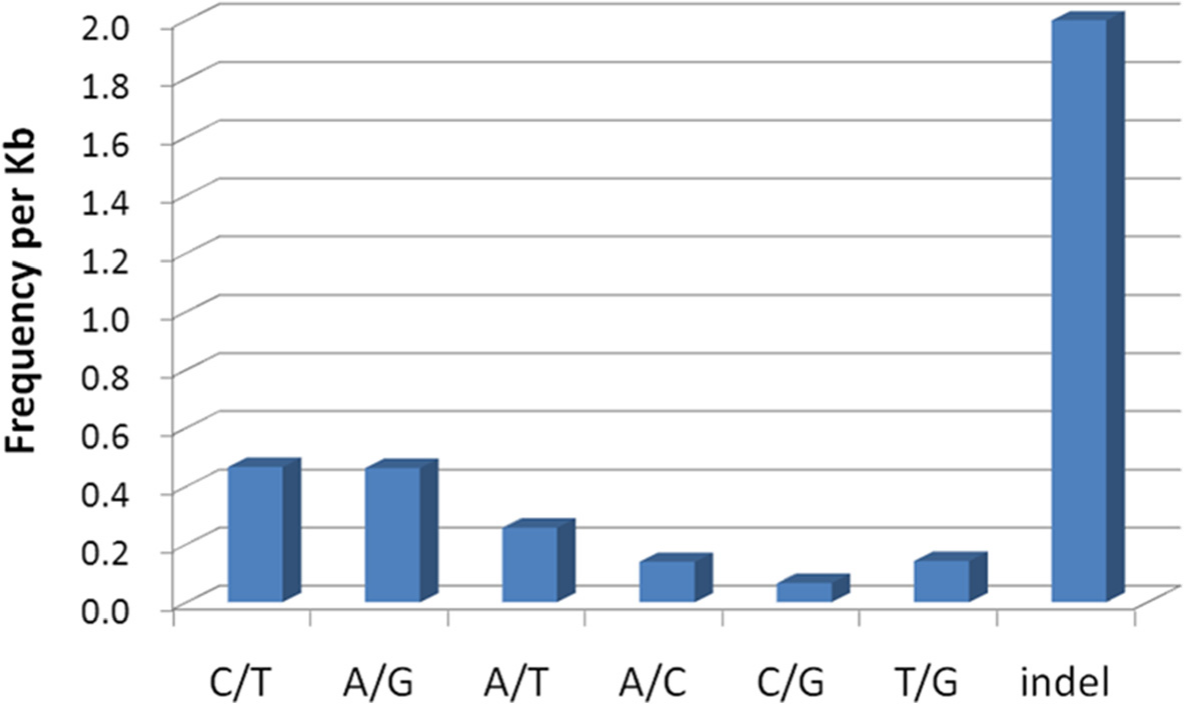
**Classification of single nucleotide polymorphisms (SNPs) identified from 454 sequences.** The overall frequency of these SNP types in *C. nobilis* transcriptome is one per 278 bp.

SNPs in *C. nobilis* EST isotigs were identified using the ssahaSNP program (http://www.sanger.ac.uk). Of the 71,719 SNPs detected, 43,433 were putative transitions (Ts) and 28,286 were putative transversions (Tv), giving a mean Ts: Tv ratio of 1.54: 1.00 across the transcriptome (Figure 4, Additional file 4: Table S4). The Ts: Tv ratio can help to identify genes affected by selection. A total of 96,320 INDELs across the transcriptome were detected. However, much caution must be paid to those INDELs because of technical problems associated with 454 pyrosequencing [39].

The overall frequency of all types of SNPs in the transcriptome, including INDELs, was one per 278 bp. Of the predicted SNPs, including INDELs, 122,927 (73.15%) were identified from isotigs covered by ten or more reads, suggesting the majority of SNPs identified in this study were covered at sufficient sequencing depth and more likely represent ‘true’ SNPs [40]. Among the SNPs, 53,831 (32.03%) were identified from isotigs with annotation information.

Twenty five of these predicted SNPs were randomly selected for validation using PCR and Sanger sequencing, and 17 of these tests (68%) were successful (Additional file 5: Table S5). The result here confirmed that the majority of computationally predicted SNPs from the 454 transcriptome sequences would benefit us in our future genetic markers development.

### Identification of carotenoid-based coloration transcripts from 454 sequences

Full length protein sequences of the 15 known genes responsible for carotenoid absorption, binding or carotenoids cleavage were used to perform tBlastn searches against 454-derived sequences. A total of 44 isotigs and 20 singletons from 454-derived sequences were identified with scores more than or equal to 100 and E values less than or equal to 1e-10, and 48 non-redundant sequences were developed and used to identify their putative functions by BlastX searches against the GenBank databases (Additional file 6: Table S6). After BlastX searches against Nr database, 26 transcripts were considered as tentative *C. nobilis* carotenoid-related transcripts (Additional file 6: Table S6).

**Figure 5:**
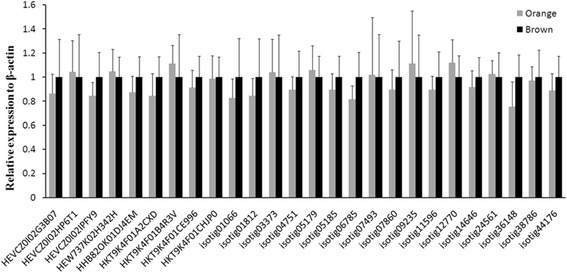
**Comparison of the expression level of 26 selected tentative carotenoid deposition transcripts in orange and brown scallop adductor muscle.** 6 scallops were used in the experiment and each expression analysis was also performed in two independent experiments. Significant difference was performed by one-way ANOVA test (*P* < 0.05).

Quantitative real-time reverse transcription PCR was performed for each of the 26 candidate transcripts to determine their relative levels of expression in the adductor muscle taken from orange scallop and brown scallop. However, we failed to find any transcripts with significant difference in expression between the orange and white adductor muscle (Figure 5). We speculated sequence variations might exist in these transcripts.

Members of three gene families, SRB/CD36, StAR/MLN64, and BCMO/BCDO have been implicated in uptake and deposition of carotenoids in animal tissues, providing plausible candidates for carotenoids accumulation. To find out whether sequence mutations existed in transcripts that belonged to these three families, we cloned 4 SRB-like genes, 2 STAR-like genes and 2 BCMO-like genes based on transcriptome data by RACE PCR (Additional file 7: Table S7), and screened possible mutation sites of CDS. Several missense mutations were found, which, however, showed no correlation with carotenoids accumulation (data not shown).

**Figure 6:**
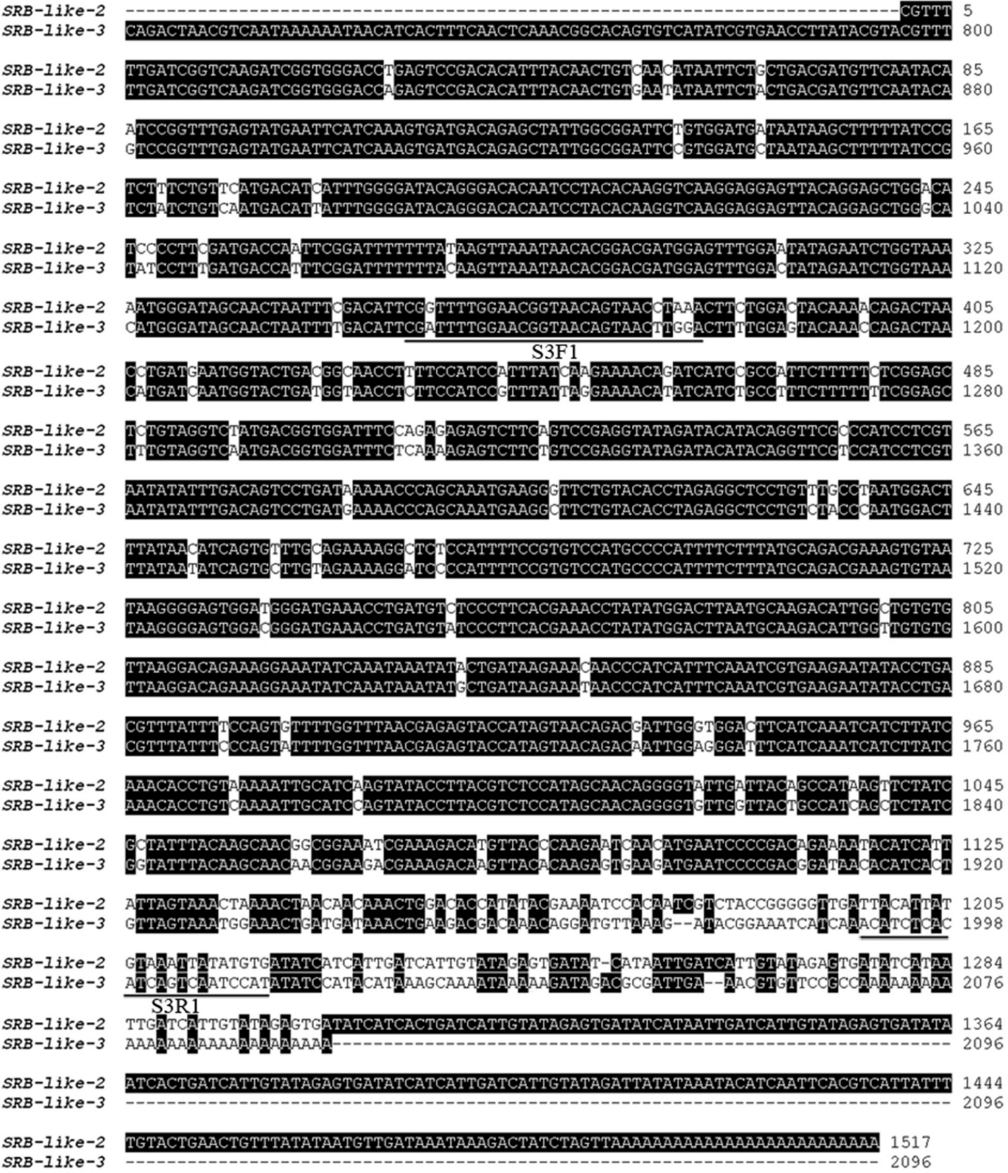
**Alignment of SRB-like-2 and SRB-like-3.** Primers S3F1 and S3R1 give special PCR amplification of SRB-like-3.

**Figure 7:**
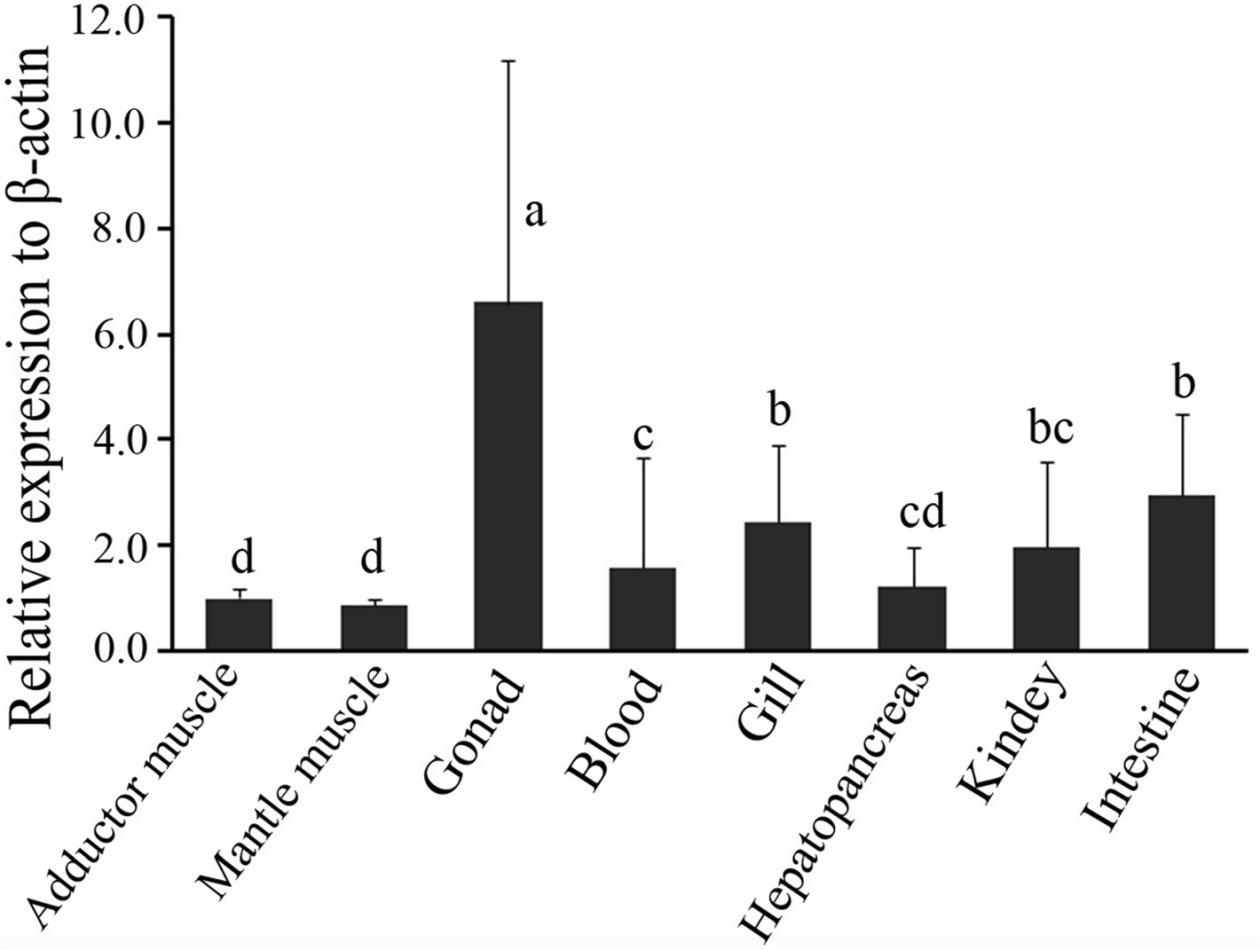
**Tissue expression profile of SRB-like-3 in orange scallop.** Different letter means significant difference by one-way ANOVA test (*P* < 0.05).

Nonetheless, a scavenge receptor gene termed SRB-like-3 was identified, which showed a high sequence similarity with SRB-like-2 (Figure 6). PCR was performed using primers (S3F1 and S3R1 shown in Figure 6) for SRB-like-3. Interestingly, SRB-like-3 was only detected in orange scallop and absent in brown scallop, suggesting that SRB-like-3 is significantly associated with high carotenoid content and possibly an important candidate gene in carotenoid deposition (Table 6). Tissue expression profile (Figure 7) showed that SRB-like-3 was highly expressed in the gonad (having highest carotenoid content) and intestine (where carotenoids were absorbed), indicating that SRB-like-3 mainly functions in these two tissues.

**Table 6:** **Progeny testing of the four lines in scallop** ***C. nobilis***

**No.**	**Carotenoid content of adductor muscle (μg/g dry weight) and SRB-like-3 detected**							
	**Line1**		**Line2**		**Line3**		**Line4**	
	**Orange**	**Brown**	**Orange**	**Brown**	**Orange**	**Brown**	**Orange**	**Brown**
1	84.86^D^	2.92^ND^	104.66^D^	5.67^ND^	89.54^D^	5.14^ND^	97.53^D^	5.24^ND^
2	90.52^D^	3.73^ND^	95.26^D^	4.96^ND^	92.56^D^	8.52^ND^	95.78^D^	5.74^ND^
3	95.77^D^	4.16^ND^	100.19^D^	5.32^ND^	109.93^D^	5.58^ND^	89.12^D^	4.24^ND^
4	86.19^D^	2.93^ND^	88.44^D^	5.32^ND^	106.01^D^	4.92^ND^	94.25^D^	5.33^ND^
5	93.48^D^	2.54^ND^	83.69^D^	4.47^ND^	93.65^D^	4.76^ND^	97.17^D^	4.60^ND^
6	96.10^D^	2.53^ND^	105.33^D^	5.05^ND^	88.54^D^	5.38^ND^	95.01^D^	5.29^ND^
7	87.46^D^	3.04^ND^	104.96^D^	5.24^ND^	103.14^D^	5.96^ND^	87.49^D^	6.26^ND^
8	97.38^D^	2.92^ND^	90.12^D^	7.03^ND^	97.54^D^	5.62^ND^	101.43^D^	5.53^ND^
9	85.77^D^	2.65^ND^	90.97^D^	5.78^ND^	92.99^D^	4.88^ND^	Missed	7.79^ND^
10	92.09^D^	3.62^ND^	Missed	5.13^ND^	108.23^D^	7.61^ND^	Missed	6.41^ND^
Mean ± SD	90.96 ± 4.69^**^	3.10 ± 0.55	95.96 ± 8.13^**^	5.40 ± 0.68	98.21 ± 7.97^**^	5.84 ± 1.25	94.72 ± 4.54^**^	5.64 ± 1.00
Total detected	10	0	9	0	10	0	8	0

^D^SRB-like-3 was detected; ^ND^SRB-like-3 was not detected. ^**^indicate very significant differences (*P* < 0.01) in carotenoid content between orange and brown scallops in the same line.

### mRNA expression of SRB-lile-3 after RNAi

**Figure 8:**
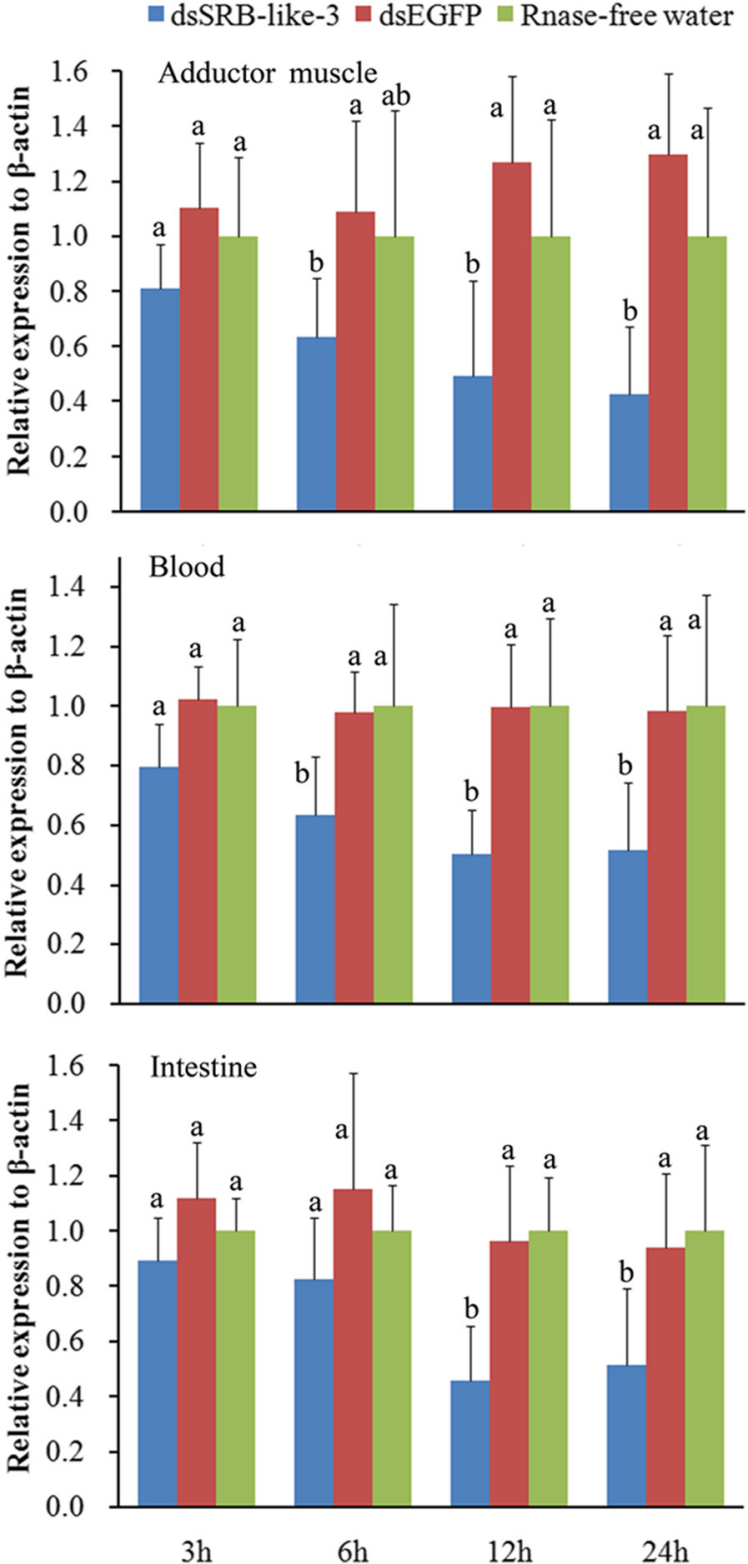
**mRNA expression of SRB-like-3 after injection of dsRNA.** Letter indicates comparison of the same tissue. Different letter means significant difference by one-way ANOVA test (*P* < 0.05).

To find out whether SRB-like-3 was involved in carotenoid deposition, dsRNA of SRB-like-3 (dsSRB-like-3) was synthesized and injected into orange scallop adductor muscle. Results of Real-Time PCR showed that the mRNA level of SRB-like-3 in tested tissue was down-regulated by dsSRB-like-3 (Figure 8). The expression level of SRB-like-3 mRNA was significantly suppressed by 67%, 48% and 45% in the adductor muscle, blood and intestine, respectively, when compared to that of the dsEGFP injected group 24 h after injection.

### Carotenoid content in the blood and adductor muscle after RNAi

**Figure 9:**
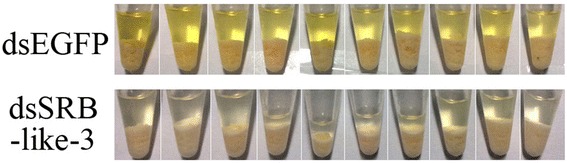
**Carotenoids extration from the blood (dsEGFP: dsRNA of EGFP; dsSRB-like-3: dsRNA of SRB-like-3).**

Total caronoid content in the blood was measured after RNAi. Color density of the dsSRB-like-3 group is lighter than that of dsEGFP group (Figure 9), implying that the dsSRB-like-3 group had a relative low carotenoid content. Carotenoid content in the blood was measured, showing that the dsSRB-like-3 group indeed had a significantly lower carotenoid content when compared to that of the dsEGFP group or blank group (Table 7), while there was no statistical difference between the dsEGFP group and the RNase-free water group. Carotenoid content in the adductor muscle showed no remarkable difference among the dsSRB-like-3, dsEGFP and Rnase-free water group (Table 8). Anyhow, our result providing compelling evidence that SRB-like-3 was a candidate gene that was at least responsible for blood carotenoid content, implying that SRB-like-3 might take part in absorption of carotenoid to blood.

**Table 7:** **Carotenoid content (CC) of blood**

**dsSRB-like-3**		**dsEGFP**		**RNase-free water**	
**ID**	**CC (μg/ml)**	**ID**	**CC (μg/ml)**	**ID**	**CC (μg/ml)**
I1	0.39	C1	1.30	W1	0.98
I2	0.28	C2	0.98	W2	1.28
I3	0.57	C3	1.13	W3	1.19
I4	0.24	C4	1.10	W4	1.30
I5	0.31	C5	1.01	W5	1.02
I6	0.51	C6	1.08	—	—
I7	0.58	C7	0.91	—	—
I8	0.43	C8	1.00	—	—
I9	0.61	C9	0.90	—	—
I10	0.37	C10	1.08	—	—
Mean ± SE	0.43^b^ ± 0.13	Mean ± SE	1.05^a^ ± 0.12	Mean ± SE	1.15^a^ ± 0.15

Note: Means with different sub letter indicate significant difference (*P* < 0.05).

**Table 8:** **Carotenoid content (CC) of adductor**

**dsSRB-like-3**		**dsEGFP**		**RNase-free water**	
**ID**	**CC (μg/ml)**	**ID**	**ID**	**CC (μg/ml)**	**ID**
I1	89.68	C1	92.16	W1	106.14
I2	95.63	C2	83.85	W2	96.50
I3	105.3	C3	114.05	W3	89.37
I4	89.95	C4	84.91	W4	98.35
I5	103.80	C5	96.81	W5	99.61
I6	96.45	C6	117.99	—	—
I7	90.77	C7	96.35	—	—
I8	107.15	C8	96.83	—	—
I9	106.14	C9	105.16	—	—
I10	104.73	C10	84.24	—	—
Mean ± SE	98.96 ± 7.21	Mean ± SE	97.23 ± 12.03	Mean ± SE	97.99 ± 6.05

Note: No significant difference was detected among the three groups.

## Conclusion

Here we documented a large-scale, multi-organ transcriptome for the noble scallop *C. nobilis*, which has the unique characterization of carotenoid accumulation but few molecular knowledge has been available. Our findings provide a nearly complete description of the expressed genes, which is a substantial contribution to the existing sequence resources for this species. Application of these resources will greatly enhance future genetic and genomic studies on scallop and other mollusks. The description of the expressed genes and their functions was illustrated according to annotation and GO assignment. 3,844 SSRs and over 120,000 high confidence variants (SNPs and INDELs) were identified that can be useful for mapping and QTLs in this scallop and related species. The most important point is that a scavenge receptor termed SRB-like-3 is only expressed in orange scallop but absent in brown scallop, significantly associated with high carotenoid content, suggesting SRB-like-3 is possibly a candidate gene responsible for carotenoid deposition in orange scallop. Results from RNAi study of this gene provides convincing evidence that SRB-like-3 is involved in carotenoid deposition in blood.

## Additional files

Additional file 1: Table S1. Sequences with significant BlastX matches against Swiss-Prot and Nr database. Additional file 2: Table S2. Gene Ontology of *C. nobilis* unique sequences. Additional file 3: Table S3. Details of SSR motifs in *C. nobilis* unique sequences. Additional file 4: Table S4. Details of SNPs and Indels in *C. nobilis* isotigs. Additional file 5: Table S5. Validation of predicted SNPs by PCR and re-sequencing. Additional file 6: Table S6. Candidate transcripts. Additional file 7: Table S7. Information of 8 candidate carotenoid deposition genes.
